# Mitochondrial Ca^2+^ flux modulates spontaneous electrical activity in ventricular cardiomyocytes

**DOI:** 10.1371/journal.pone.0200448

**Published:** 2018-07-12

**Authors:** An Xie, Anyu Zhou, Hong Liu, Guangbin Shi, Man Liu, Kenneth R. Boheler, Samuel C. Dudley

**Affiliations:** 1 Department of Medicine, Lillehei Heart Institute, University of Minnesota, Minneapolis, MN, United States of America; 2 Stem Cell and Regenerative Medicine Consortium, LKS Faculty of Medicine, Hong Kong University, Hong Kong, P.R. China; Rush University Medical Center, UNITED STATES

## Abstract

**Introduction:**

Ca^2+^ release from sarcoplasmic reticulum (SR) is known to contribute to automaticity via the cytoplasmic Na^+^-Ca^2+^ exchanger (NCX). Mitochondria participate in Ca^2+^ cycling. We studied the role of mitochondrial Ca^2+^ flux in ventricular spontaneous electrical activity.

**Methods:**

Spontaneously contracting mouse embryonic stem cells (ESC)-derived ventricular cardiomyocytes (CMs) were differentiated from wild type and ryanodine receptor type 2 (RYR2) knockout mouse ESCs and differentiated for 19–21 days. Automaticity was also observed in human induced pluripotent stem cell (hiPSC)-derived ventricular CMs differentiated for 30 days, and acute isolated adult mouse ventricular cells in ischemic simulated buffer. Action potentials (APs) were recorded by perforated whole cell current-clamp. Cytoplasmic and mitochondrial Ca^2+^ transients were determined by fluorescent imaging.

**Results:**

In mouse ESC-derived ventricular CMs, spontaneous beating was dependent on the L-type Ca^2+^ channel, cytoplasmic NCX and mitochondrial NCX. Spontaneous beating was modulated by SR Ca^2+^ release from RYR2 or inositol trisphosphate receptors (IP_3_R), the pacemaker current (I_f_) and mitochondrial Ca^2+^ uptake by the mitochondrial Ca^2+^ uniporter (MCU). In RYR2 knockout mouse ESC-derived ventricular CMs, mitochondrial Ca^2+^ flux influenced spontaneous beating independently of the SR Ca^2+^ release from RYR2, and the mitochondrial effect was dependent on IP_3_R SR Ca^2+^ release. Depolarization of mitochondria and preservation of ATP could terminate spontaneous beating. A contribution of mitochondrial Ca^2+^ flux to automaticity was confirmed in hiPSC-derived ventricular CMs and ischemic adult mouse ventricular CMs, confirming the findings across species and cell maturity levels.

**Conclusions:**

Mitochondrial and sarcolemma NCX fluxes are required for ventricular automaticity. Mitochondrial Ca^2+^ uptake plays a modulatory role. Mitochondrial Ca^2+^ uptake through MCU is influenced by IP_3_R-dependent SR Ca^2+^ release.

## Introduction

Adult ventricular cardiomyocytes (CMs) show spontaneous electrical activity known as abnormal automaticity when injured. This spontaneous activity is thought to contribute to arrhythmic risk. Mouse embryonic stem cell (ESC)-derived and human induced pluripotent stem cell (hiPSC)-derived ventricular-like CMs show similar spontaneous electrical activity that allows for more detailed the study of the ion currents underlying this ventricular automaticity. In these cells, spontaneous electrical activity is modulated strongly by sarcoplasmic reticulum (SR) Ca^2+^ release through ryanodine receptor type 2 (RYR2). This Ca^2+^ release activates the Na^+^/Ca^2+^ exchanger (NCX) to produce an inward current that can accelerate the rate of spontaneous membrane depolarization[1–4]. Targeted inactivation of the NCX type 1 results in the lack of a spontaneous heart beat in NCX type 1-null embryos[5], making Ca^2+^ cycling a critical requirement of spontaneous electrical activity in whole hearts. Other ion channels and receptors that are involved in spontaneous electrical activity include the inward rectifier potassium current (I_K1_)[6], the I_f_ pacemaker current[7], and the inositol trisphosphate receptor (IP_3_R)-mediated SR Ca^2+^ release[8]. These mechanisms of automaticity in ESC-derived working CMs are similar to those in the cardiac pacemaker cells in the terms of the “M-clock (voltage clock)” and the “Ca^2+^-clock”[9, 10].

Mitochondria are involved in the regulation of cellular Ca^2+^. It has been reported that mitochondria Ca^2+^ uptake contributes to buffering cytoplasmic Ca^2+^ peaks in cardiomyocytes[11]. Mitochondrial Ca^2+^ cycling modulates cardiac pacemaker and atrial cell automaticity[12, 13]. Nevertheless, the role of mitochondrial Ca^2+^ cycling in automaticity of cardiac ventricular cells is unclear. Mitochondrial Ca^2+^ homeostasis involves Ca^2+^ influx mainly through the mitochondrial Ca^2+^ uniporter (MCU) driven by the mitochondrial membrane potential (Ψm)[14]. Other mitochondrial Ca^2+^ uptake pathways include the ryanodine receptor type 1[15]. Mitochondrial Ca^2+^ efflux occurs mainly through the mitochondrial NCX[16].

Because of the role of spontaneous electrical activity in initiating ventricular arrhythmias, we tested whether mitochondrial Ca^2+^ flux influenced ventricular CM spontaneous electrical activity in the presence and absence of the SR Ca^2+^ release channel, using ESC-derived mouse ventricular CMs. Results were confirmed in adult mouse ventricular and hiPSC-derived CMs to attempt to ensure applicability of the results to human pathophysiology.

## Materials and methods

All relevant data are available from the Figshare repository at the following DOI: 10.6084/m9.figshare.6260555.

### Culture and differentiation of mouse ESCs and hiPSC

The mouse R1 ESC cell line transfected with a neomycin resistance gene (ATCC, Manassas, VA, USA) was used throughout the present study. To knock out RYR2, gene targeting of the R1 cells was performed by sequential homologous recombination of the two chromosomal alleles of RYR2 in R1 ESCs. PCR analysis and Western blotting of ESCs, embryonic bodies, and CM-derived RNA or protein confirmed no evidence of RYR2 transcript or protein, respectively[3]. Undifferentiated ESCs were maintained and expanded as described previously[17–19]. A suspension containing about 500 ESCs in 30 μL media enriched in FBS (20%) was placed as hanging drops on the lids of petri dishes filled with 10 mL PBS. After two days in hanging drops, embryoid bodies were removed and plated onto 0.1% gelatin-coated petri dishes. The 7 day old embryoid bodies were used for the final stages of CM differentiation. Usually after 9–12 days of plating, embryoid bodies showed areas of spontaneous beating. Embryoid bodies differentiated after 18 days were used to isolate single CMs. Eight to ten embryoid bodies containing spontaneously beating areas were used for CM isolation using a procedure described previous. The isolated CMs were plated on 35 mm dishes coated with 0.1% gelatin and 20 μg/mL laminin in cultivation medium and incubated in 37°C. After overnight incubation, spontaneously beating CMs were used for electrophysiological studies within 1–3 days after the cell isolation.

The hiPSC-derived CMs (iCell^®^ Cardiomyocytes^2^) differentiated for 30 days were purchased from Cellular Dynamics International, Inc. (Madison, WI, USA). Cells were plated and maintained according to the manufacturer’s instructions. Ventricular CMs were selected according to the action potential (AP) morphology[20].

### Isolation and induction of abnormal automaticity in adult mouse ventricular CMs

All animal protocols were in accordance with the guidelines of the Animal Care and Use Committee of the University of Minnesota and conformed to the Guide for the Care and Use of Laboratory Animals published by the National Institutes of Health. Adult mouse ventricular CMs were isolated from 11–12 weeks old C57BL/6 mice (22–25 g; Charles River, Wilmington, MA) as described. Randomly selected mice of either sex were anaesthetized using inhaled isoflurane (3% for induction and 1.5–2% for maintenance). After the anesthetized mouse was non-responsive to toe pinch, a thoracotomy was performed. The rib cage was cut bilaterally and the heart exposed. The heart was then excised and placed immediately in a petri dish filled with ice cold Tyrode’s solution. The heart was then perfused with buffer (in mmol/L: NaCl/ 113, KCl 4.7, Na_2_HPO_4_ 0.6, KH_2_PO_4_ 0.6, MgSO_4_ 1.2, Phenol Red 0.032, NaHCO3 12, KHCO_**3**_ 10, HEPES 10, Taurine 30, and 2-3-butanedione monoxime 10) and digested with collagenase II (Worthington Biochemical Co. Lakewood, NJ). CMs were washed with control buffers (in mmol/L: NaCl 133.5, KCl 4, Na_2_HPO_4_ 1.2, HEPES 10, and MgSO_4_ 1.2) with serially increasing Ca^2+^ concentrations (0.2, 0.5, and 1 mmol/L). Then, myocytes were incubated in MEM medium (modified Eagle's medium with 1% insulin–transferrin–selenium, 0.1% bovine serum albumin, 1% l-glutamine, and 1% penicillin/streptomycin) in a 95% O_2_/5% CO_2_ incubator at 37°C for 1 h prior to being used for Ca^2+^ transient measurements. During experiments, the acute isolated mouse ventricular CMs were exposed to a simulated ischemic buffer containing (in mM: 110 NaCl, 10 KCl, 1 MgSO_4_, 10 Na^+^-HEPES, 2.5 CaCl_2_, 20 2′-deoxyglucose, 20 Na^+^-lactate and 1 NaCN, at pH 6.6) at 37°C for 10 min to induce spontaneous beating[21].

### AP recordings from the ESC-derived CMs

APs were recorded at using perforated whole-cell current-clamp as described before[1]. To measure spontaneous APs, the culture medium was replaced with Tyrode solution contained (in mmol/L) 140 NaCl, 5.4 KCl, 1 MgCl_2_, 10 HEPES, 1.8 CaCl_2_, 0.33 NaH_2_PO_4_ and 5.5 glucose (pH 7.4). Glass patch pipettes (World Precision Instrument, Sarasota, FL, USA) were pulled to a resistance of 2–5 MΩ. For AP measurements, the intracellular solution consisted of (in mmol/L) 120 potassium gluconate, 20 KCl, 5 NaCl, 5 HEPES, and 5 MgATP (pH 7.2). APs were recorded using perforated whole-cell current-clamp and an Axopatch-200B amplifier (Molecular Devices, Sunnyvale, CA, USA). Membrane potential recordings performed in the current-clamp configuration were low pass filtered at 10 kHz and digitized at 20 kHz using a gap-free acquisition mode. For perforated-patch experiments, β-escin (50 μmol/L) was added to pipette solution. Only CMs with a ventricular-like AP were further studied. Criteria for selection of Wt ventricular CMs were a resting membrane potential (<-70 mV) and AP duration at 90% repolarization (> 180 ms)[18].

### Intracellular Ca^2+^ measurements

To visualize changes in the intracellular Ca^2+^ transients, mouse ESC-derived CMs and acutely isolated mouse ventricular CMs were loaded with Indo-1 AM (ThermoFisher Scientific, Minneapolis, MN, USA 2 μmol/L) for 40 min followed by 20 min of de-esterification at room temperature[22]. The CMs were transferred onto the stage of a real-time fluorescence microscope (IonOptix LLC, Milton, MA, USA). Ca^2+^ transients were acquired at a rate of 1 kHz. Analysis of the signals was performed using IonWizard. Indo-1was excited at 405 nm and emitted fluorescence was collected at wavelengths of 470–490 nm. Selective experiments using Fluo-4 as the fluorescent reporter with mESC-derived cardiomyocytes showed comparable results to Indo-1 (data not shown). Fluo-4 AM (ThermoFisher Scientific), 3 μmol/L, was loaded in hiPSC-derived CMs for 20 min followed with 20 min de-esterification. The excitation wavelength was 488 nm and the absorption wavelength was 500 nm. Cells were transferred onto the stage of a real-time florescence microscope (Olympus IX81, Japan). Analysis of the signals was performed with the software MetaMorph (version 7.8.11.0, Nashville, TN). Cytoplasmic Ca^2+^ transients were presented as the ratio of intensities normalized by background fluorescence (F/F_0_).

### Mitochondrial Ca^2+^ recordings

Mitochondrial Ca^2+^ transients were monitored by loading cells with Rhod-2 AM (ThermoFisher Scientific, 1 μM, load for 1 h at 37°C), washing the cells twice, and allowing for de-esterification for 1 h in Rhod-2-free DMEM. In these measurements, the ruptured current-clamp technique was used with 20 μM EGTA added in the pipette solution. Minor cytosolic traces of Rhod-2 were eliminated by whole-cell dialysis by the pipette solution[23, 24]. We used very big glass pipette to do this experiment with a resistance ~2.5 MΩ. This helps a lot to reduce the cytoplasmic Ca^2+^ contaminate. Rhod-2 intensity was sampled at a rate of 1 kHz. Mitochondrial Ca^2+^ transients were presented as background-subtracted normalized fluorescence (F/F_0_).

### Silencing MCU

To silence MCU, specific MCU siRNAs were synthesized by Life Technologies[25]. siRNA-MCU1 targets nucleotides 899–917 of the corresponding mRNA (5’-GCCAGAGACAGACAAUACUtt-3’ and 3’-ttCGGUCUCUGUCUGUUAUGA-5’. siRNA-MCU2 targets nucleotides 360–378 of the corresponding mRNA (5’-GGGAAUUGACAGAGUUGCUtt-3’ and 3’-ttCCCUUAACUGUCUCAACGA-5’). The non-targeting siRNA (scrambled) was used as a control (5’-GCCUAAGAACGACAAAUCAtt-3’ and 3’-ttCGGAUUCUUGCUGUUUAGU-5’). Cells were transfected by Lipofectamine RNAiMAX reagent (ThermoFisher Scientific) for 72 h with a siRNA concentration of 30 pmol/L. MCU mRNA was measured after 72 h by qPCR. Single Cell-to-CT^TM^ Kit (PN 4458237) and primers (MCU primers: Mm01168773_m1, Cat.# 4351372; β-actin primers: Mm00607939_s1, Cat.# 4331182) were from ThermoFisher Scientific.

All data are presented as mean ± SE. The t-tests and Chi-tests were employed for statistical analysis. All experiments were carried out at 37°C.

## Results and discussion

Ventricular spontaneous electrical activity or abnormal automaticity is thought to be a cause of arrhythmias, especially after ischemic insult[26]. Automaticity is thought to be dependent on sarcolemmal and SR Ca^2+^ oscillations. For example, RYR2 loss contributes to bradycardia, arrhythmia, and sudden death in adult mice[27]. Major ion channels contributors to automaticity include the cardiac Na^+^ channel, L-type Ca^2+^ channel, T-type Ca^2+^ channel, HCN channel, K^+^ channel and NCX1[28].

Mitochondria are known to participate in Ca^2+^ oscillations[12, 29]. Mitochondrial Ca^2+^ cycling modulates cardiac pacemaker and atrial cell automaticity. In pacemaker cells, inhibition of mitochondrial Ca^2+^ influx into (Ru360) or Ca^2+^ efflux from (CGP) mitochondria modulated the spontaneous AP frequency in opposing directions (111 ± 1% control or 89 ± 2% control), respectively. In atrial cells, NCLX knockdown using siRNA results in slowing of spontaneous electrical activity, an effect similar to that seen in pacemaker cells[12, 13]. Therefore, it stands to reason that mitochondria may be able to influence automaticity of ventricular CMs. Nevertheless, the mechanisms of how sarcolemmal, sarcoplasmic reticular, and mitochondrial Ca^2+^ affect spontaneous activity and whether these mechanisms are similar in pacemaker, atrial, and ventricular cardiomyocytes are unknown.

### Spontaneous electrical activity was modulated by mitochondrial Ca^2+^ cycling

**Fig 1:**
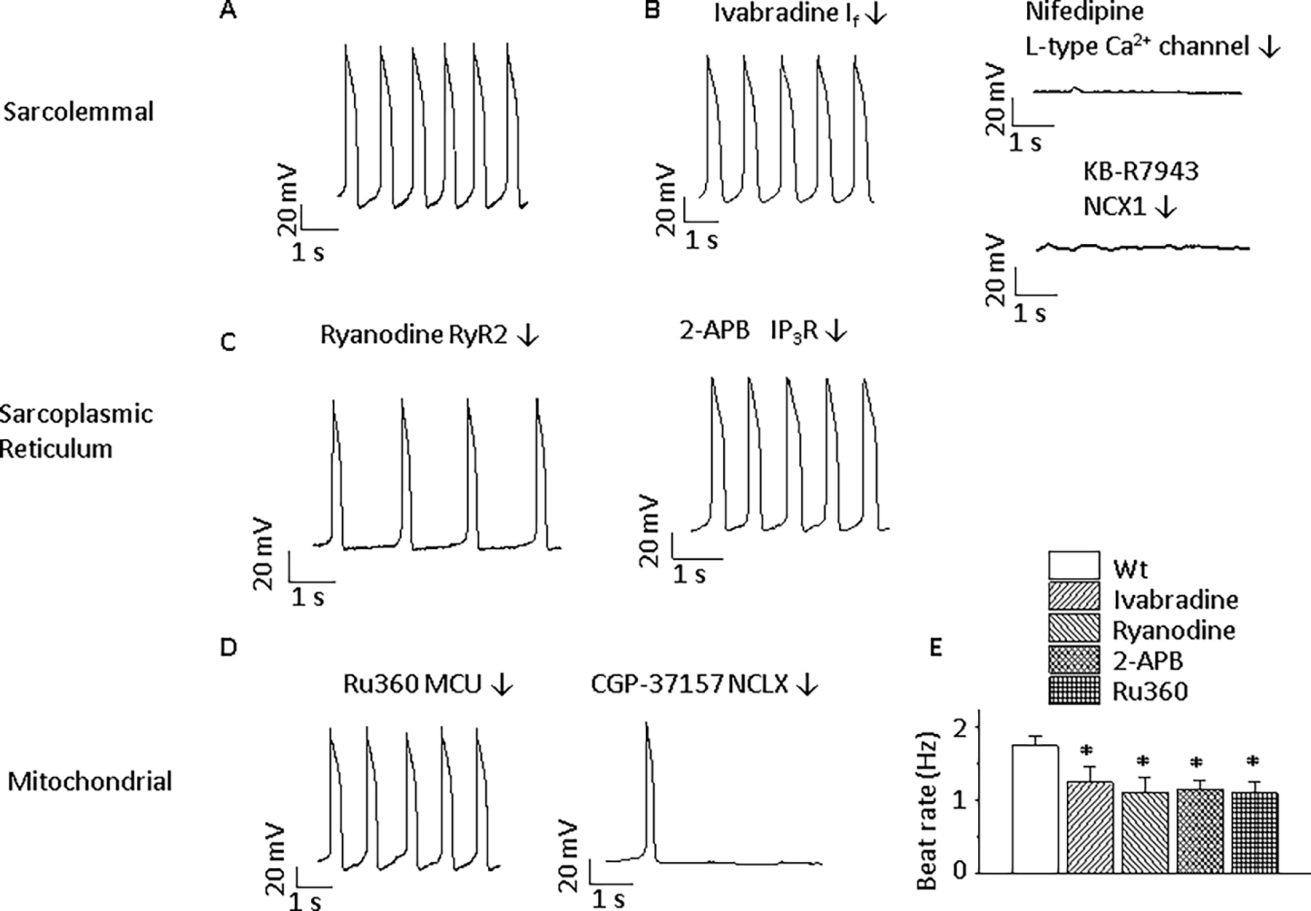
Modulation of spontaneous beating by blocking ion channels and receptors in Wt ventricular-like ESC-derived CMs. **A**) Typical APs of Wt ventricular-like ESC-derived CM recorded by perforated whole-cell current-clamp technique (n = 15). **B**) Effects of sarcolemmal ion channel or transporter blockers. Beating rate is reduced by the I_f_ specific blocker, 10 μmol/L ivabradine (n = 6). Automaticity is eliminated by the L-type Ca^2+^ channel blocker, 10 μmol/L nifedipine (n = 6), and the NCX blocker, 10 μmol/L KB-R7943 (n = 5). **C**) Effects of sarcoplasmic reticulum ion channel blockers. Spontaneous beating is modulated by the SR Ca^2+^ release blocker, 10 μmol/L Ryanodine (n = 5), and the IP_3_R blocker, 2 μmol/L 2-APB (n = 4). **D**) Effects of altering mitochondrial Ca^2+^ handling. Spontaneous beating is modulated by mitochondrial Ca^2+^ influx blocker, 1 μmol/L Ru360 (in pipette solution, n = 5), and mitochondrial NCX (NCLX) blocker, 3 μmol/L CGP-37157 (n = 6). **E**) Summary of effects on spontaneous beating. *p<0.05, compared with control group.

Wild type (Wt) ESC-derived ventricular-like CMs showed spontaneous beating (Fig 1A). Spontaneous electrical activity was absolutely dependent on sarcolemmal Ca^2+^ flux since blocking the L-type Ca^2+^ channel with 10 μmol/L nifedipine for 2 min or blocking the sarcolemmal NCX with the NCX1 specific blocker[30], KB-R7943 (10 μmol/L, for 2 min), arrested CM electrical activity (Fig 1B). While spontaneous electrical activity was dependent on L-type Ca^2+^ channel activity, it is unclear whether the L-type Ca^2+^ channel provides directly Ca^2+^ to the NCX or indirectly through the SR. Spontaneous beating was modulated by the pacemaker current through hyperpolarization-activated cyclic nucleotide (HCN)-gated channels, designated as I_f_. The specific blocker, 10 μmol/L ivabradine[31], had a modest inhibitory effect on the beating rate (from 1.7 ± 0.1 to 1.2 ± 0.2 Hz, *p*<0.05, Fig 1B and 1E) at 2 min after application. Consistent with a role for SR Ca^2+^ cycling in the modulation of spontaneous electrical activity[4, 8], the beating rate could be substantially reduced by treating with 10 μmol/L ryanodine more than 30 min, which inhibited the main SR Ca^2+^ release pathway through the RYR2, and by treating with 2 μmol/L 2-aminoethyl diphenylborinate (2-APB) for more than 15 min, which inhibited SR Ca^2+^ release pathway through IP_3_Rs. The spontaneous beating rates were greatly reduced to 54% and 71% by ryanodine and 2-APB, respectively (Fig 1C and 1E).

Mitochondrial Ca^2+^ flux also influenced spontaneous beating in Wt CMs. Inhibiting mitochondrial Ca^2+^ uptake by intracellular application of a MCU-specific blocker, 1 μmol/L Ru360, had a similar effect on beating rate as inhibiting SR Ca^2+^ release from IP_3_Rs (Fig 1D and 1E). The mitochondrial NCX blocker, 3 μmol/L CGP-37157 (CGP)[32], terminated action potential (AP) firings (Fig 1D) after 2 min of application. In order to avoid nonspecific effects of the antagonists, the concentrations used of these compounds were less than or equal to the reported concentrations that were specific for the intended targets[22, 30–34].

**Fig 2:**
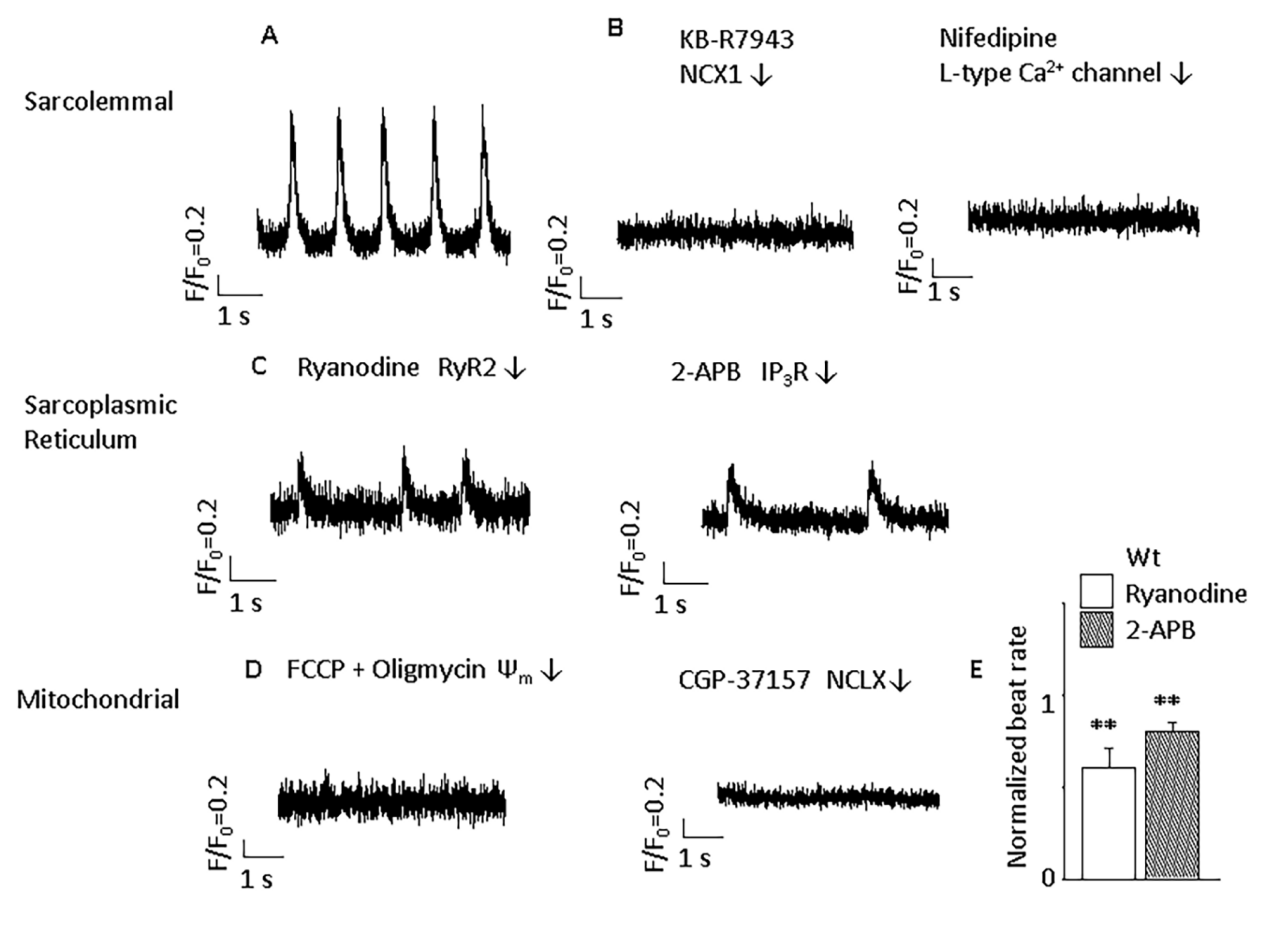
Cytosolic Ca^2+^ traces in Wt ventricular-like ESC-derived CMs. **A**) Typical cytoplasmic Ca^2+^ transients in a Wt ventricular-like CM. **B**) Cytoplasmic Ca^2+^ transients are abolished by the NCX blocker, 5 μmol/L KB-R7943 (n = 4) and the L-type Ca^2+^ channel blocker, 10 μmol/L nifedipine (n = 4). **C**) Blocking RyR and IP_3_Rs with 10 μmol/L ryanodine (n = 5) and 2 μmol/L 2-APB (n = 4), respectively, reduces but does not eliminate the cytoplasmic Ca^2+^ transients. **D**) Cytoplasmic Ca^2+^ transients are abolished by mitochondrial depolarization with 300 nmol/L FCCP and 1 μmol/L oligomycin (n = 5). Inhibiting mitochondrial Ca^2+^ release with 3 μmol/L CGP-37157 also terminates cytoplasmic Ca^2+^ transients (n = 6)**. E**) The summary of ryanodine and 2-APB’s effects on spontaneous beating. Data were normalized with those before blockers were used. **p<0.01, compared with those before compounds applied.

The experimental results of cytoplasmic Ca^2+^ transients confirmed the effects of above antagonists on the spontaneous rhythm in ESC-derived ventricular-like cells (Fig 2).

### Mitochondrial Ca^2+^ cycling affected spontaneous electrical activity in the absence of RYR2 Ca^2+^ release

**Fig 3:**
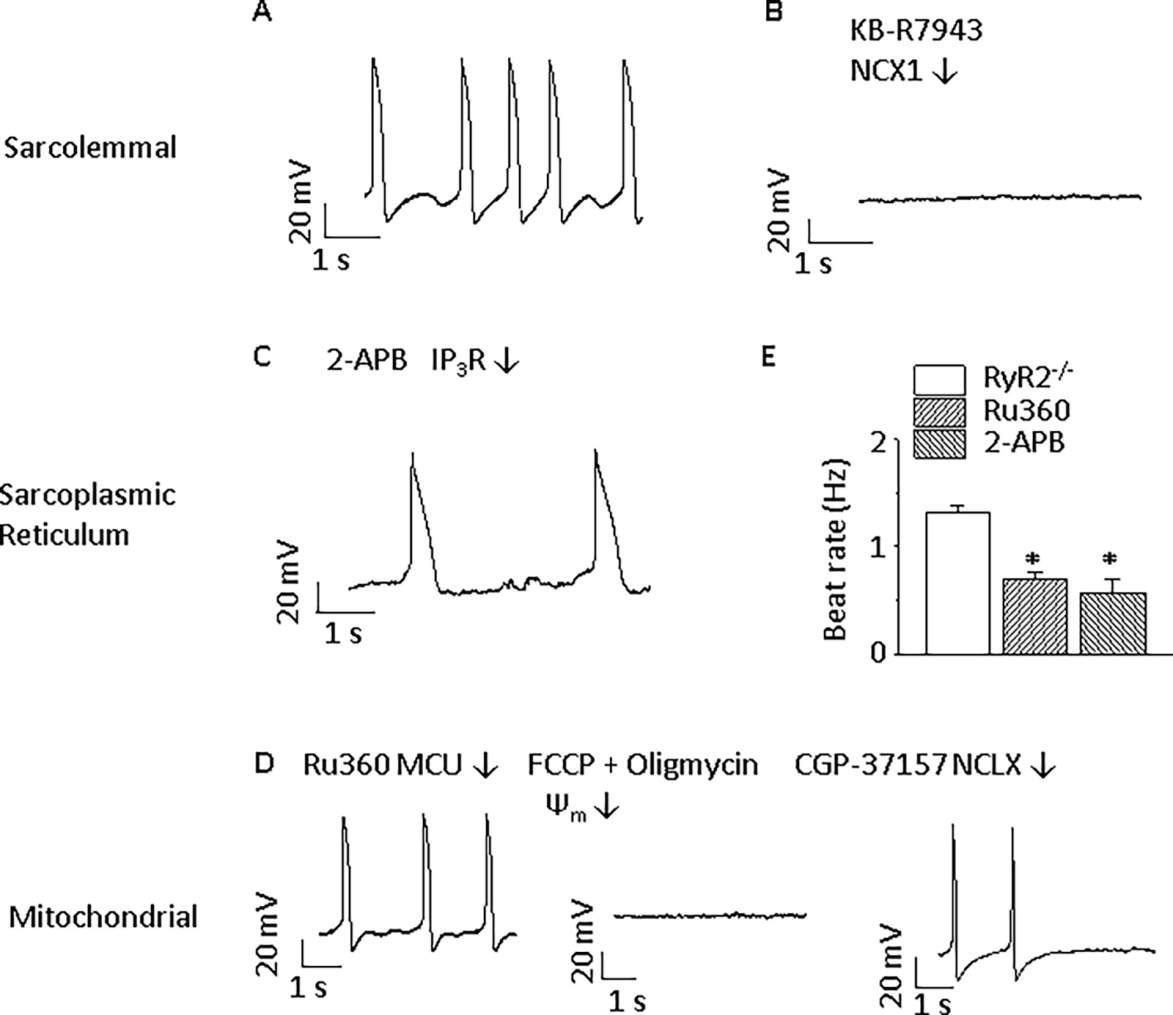
Regulation of automaticity by blocking ion channels and transporters in RYR2^-/-^ ventricular-like ESC-derived CMs. **A**) In the absence of ryanodine receptors, there is still spontaneous beating (n = 14). **B**) Effects of sarcolemmal alterations. Spontaneous beating is eliminated by the NCX blocker, 5 μmol/L KB-R7943 (n = 5). **C**) Effect of sarcoplasmic reticulum ion channel blocker. Blocking IP_3_Rs with 2 μmol/L 2-APB could inhibit spontaneous beating (n = 7). **D**) Effects of mitochondrial alterations. Spontaneous beating is modulated by mitochondrial Ca^2+^ influx blocker, 1 μmol/L Ru360 (in pipette solution, n = 7). Mitochondria depolarization with 300 nmol/L Carbonyl cyanide 4-(trifluoromethoxy)phenylhydrazone (FCCP) and 1 μmol/L oligomycin arrests spontaneous activity (n = 5). Inhibiting mitochondrial Ca^2+^ release with 3 μmol/L CGP-37157 also terminates spontaneous beating (n = 10). **E**) Summary of effects on spontaneous beating. *p<0.05 compared with control group. Ψ_m_: mitochondrial membrane potential.

**Fig 4:**
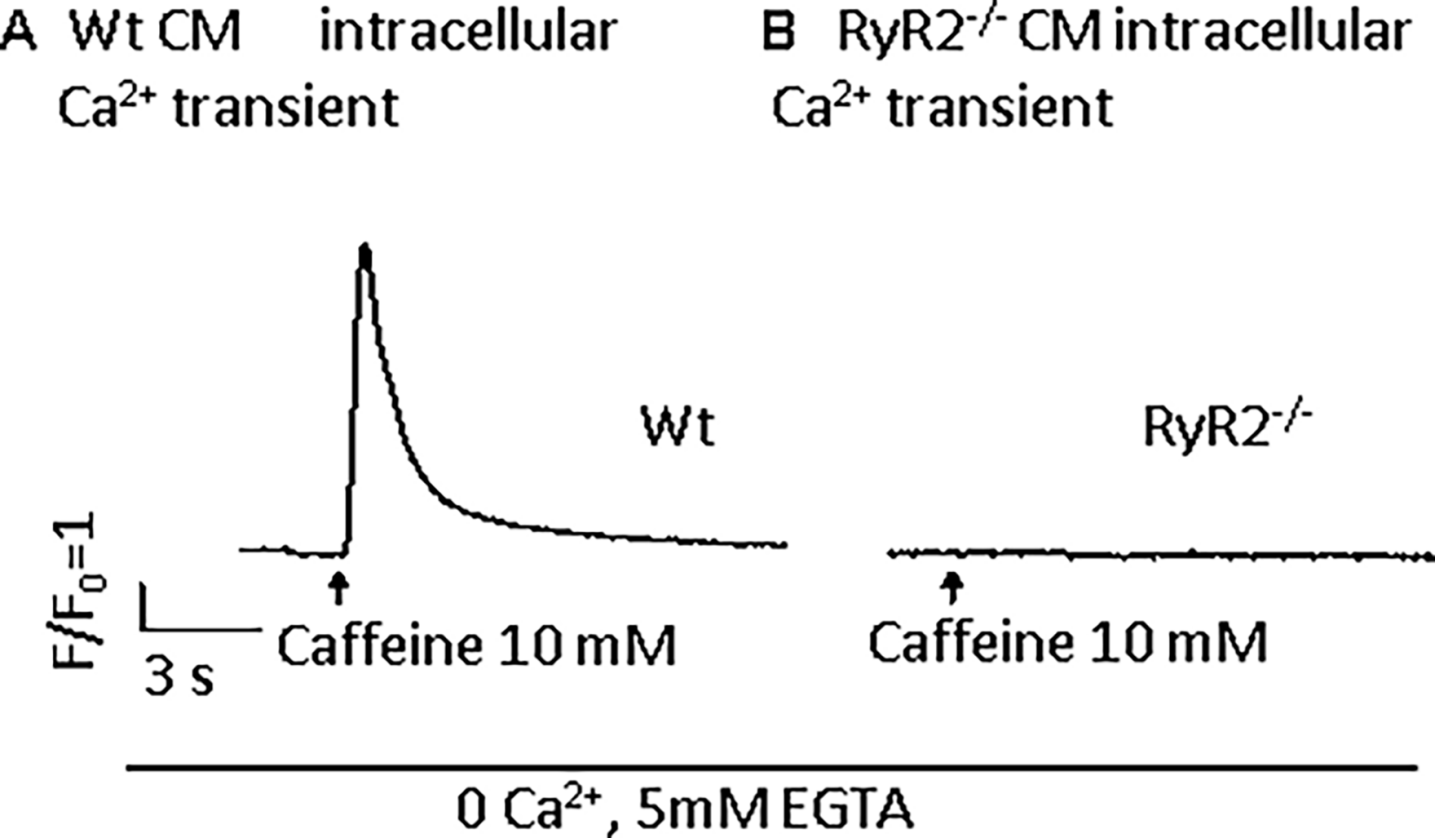
RYR2^-/-^ ventricular-like ESC-derived CMs have no Ca^2+^ release with caffeine exposure. **Left panel**: A representative Wt ventricular-like CM with Ca^2+^ release induced by 10 mol/L caffeine. **Right panel**: Caffeine (10 mmol/L) does not induce SR Ca^2+^ release from RYR2^-/-^ CMs. n = 5 for each group.

**Fig 5:**
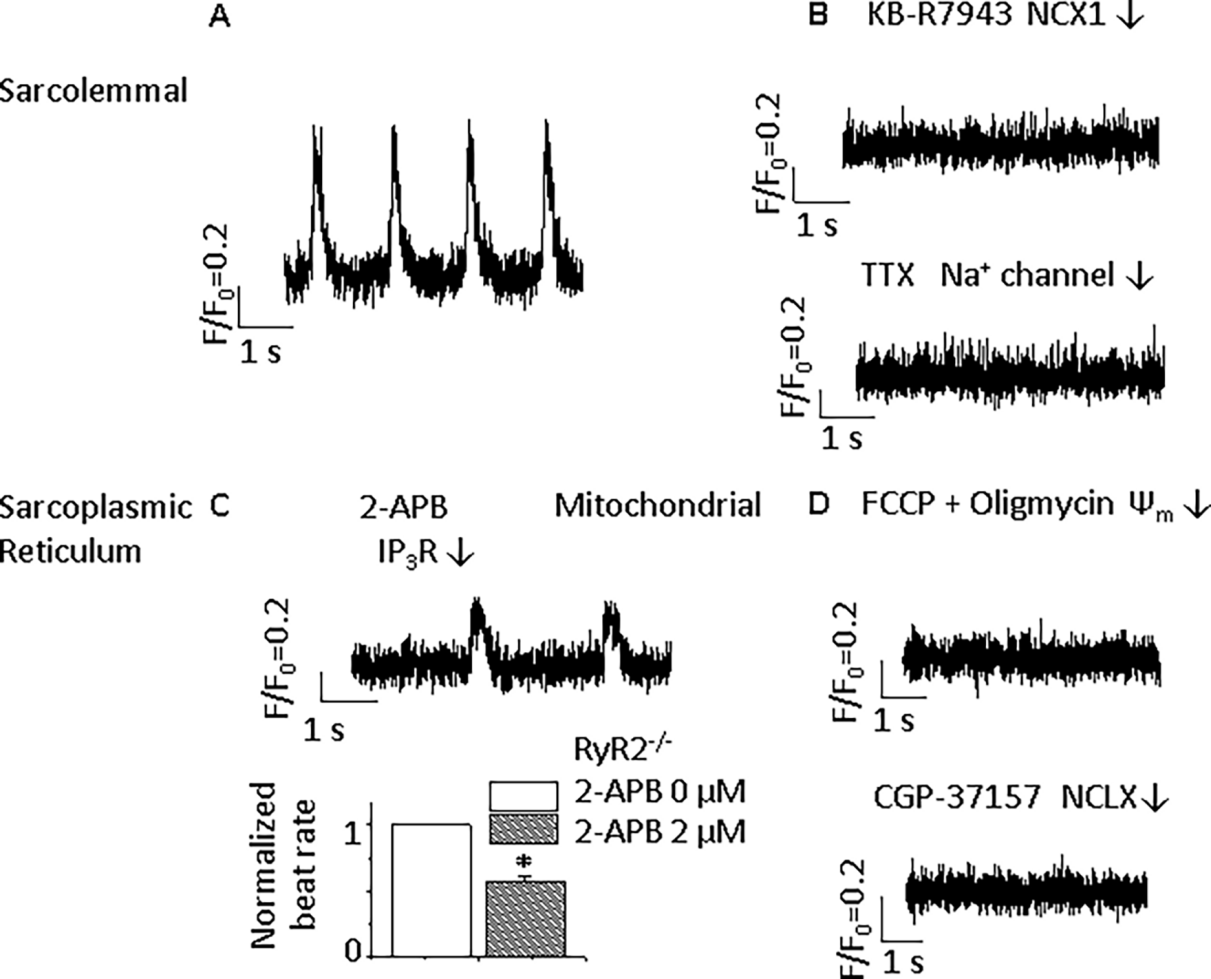
Cytoplasmic Ca^2+^ transients in RYR2^-/-^ ventricular-like ESC-derived CMs. **A**) Typical cytoplasmic Ca^2+^ transients in a RYR2^-/-^ CM. **B**) Cytoplasmic Ca^2+^ transients are eliminated by the NCX blocker, 5 μmol/L KB-R7943 and the Na^+^ channel blocker, 30 μmol/L TTX (n = 4 for each group). **C**) Blocking IP_3_Rs with 2 μmol/L 2-APB reduces but does not eliminate the cytoplasmic Ca^2+^ transients (n = 8). Data were normalized with those before blocker was used. *p<0.05, compared with those before antagonist applied. **D**) Cytoplasmic Ca^2+^ transients are abolished by mitochondrial depolarization with 300 nmol/L FCCP and 1 μmol/L oligomycin (n = 5). Inhibiting mitochondrial Ca^2+^ release with 3 μmol/L CGP-37157 also terminates cytoplasmic Ca^2+^ transients (n = 6).

In order to isolate the effect of mitochondrial Ca^2+^ cycling, we utilized cells lacking RYR2 receptor. ESC-derived ventricular-like RYR2 knockout (RYR2^-/-^) CMs showed the expected reduction in spontaneous beating similar to the effect of ryanodine on Wt CMs (Figs 1A, 1C, 3A and 3E). To demonstrate the lack of SR Ca^2+^ release, 10 mmol/L caffeine was unable to induce Ca^2+^ release from SR in RYR2^-/-^ CMs (Fig 4). TTX at a concentration of 30 μM could totally block the spontaneous beating of ESC-derived ventricular-like CMs (Fig 5B). It is known that phase 0 of the AP in ventricular cells is mediated by inward Na^+^ current. Consistent with this idea, TTX totally blocked the spontaneous beating of ESC-derived ventricular-like CMs, an effect differing from that of TTX on pacemaker cells[28].

**Fig 6:**
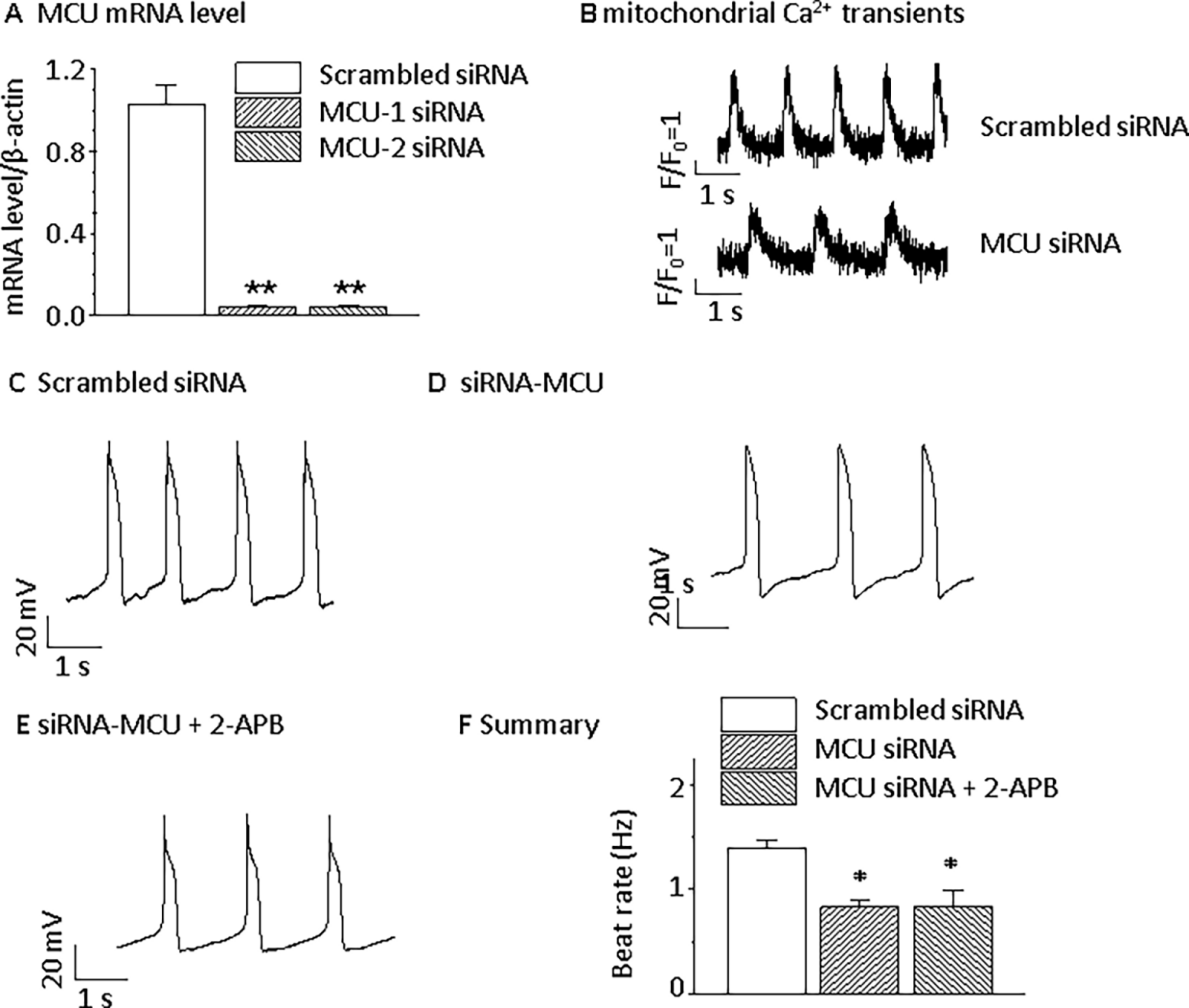
MCU knockdown alters spontaneous beating in RYR2^-/-^ ventricular-like ESC-derived CMs. **A**) MCU mRNA was decreased by 96% after either of two MCU siRNAs for 72 h. **p<0.01, compared with the scrambled siRNA group. **B**) MCU silencing by MCU-1 or MCU-2 siRNA for 72 h relative to scrambled siRNA controls results in reduced mitochondrial Ca^2+^ transients (n = 5 for each group). **C**) Representative APs recorded from a RYR2^-/-^ ventricular-like CM after scrambled siRNA transfection and incubation for 72 h (n = 14). **D**) Spontaneous beating is depressed after MCU-1 or MCU-2 siRNA silence for 72 h (n = 16). **E**) Inhibition of IP_3_R has no effect on spontaneous beating after MCU knockdown (n = 7). **F**) The summary of MCU siRNA effects on spontaneous beating. *p<0.05, compared with scrambled siRNA groups.

In ESC-derived ventricular-like RYR2^-/-^ CMs, 1 μmol/L Ru360 significantly inhibited pacemaker activity by 34.8% (Fig 3D and 3E, *p*<0.05), and Ru360 showed a similar effect to that in Wt CMs, reducing the spontaneous beating rate by 45.0% (Fig 1D and 1E). MCU silencing by MCU-1 or MCU-2 siRNA for 72 h relative to scrambled siRNA controls resulted in reduced MCU mRNA abundance by 96%, decreased mitochondrial Ca^2+^ transients, and a substantial slowing of the spontaneous beating rate (Fig 6). After MCU silencing by MCU-1 or MCU-2 siRNA for 72 h, the peak mitochondrial Ca^2+^ transient (F/F_0_) was significantly reduced from 2.7 ± 0.6 to 1.3 ± 0.4 while the Ca^2+^ transient rise time constant substantially increased from 78.8 ± 19.5 to 155.0 ± 24.4 ms and the decay time constant increased from 129.8 ± 25.4 to 224.3 ± 31.7 ms. Basal mitochondrial Ca^2+^ (F/F_0_) was slightly decreased from 2.3 ± 0.3 to 1.7 ± 0.9 (Fig 6B).

To confirm the role of mitochondrial Ca^2+^ influx in the spontaneous electrical activity, mitochondria were depolarized and ATP preserved with the combination of carbonyl cyanide 4-(trifluoromethoxy) phenylhydrazone (FCCP), a protonophoric uncoupler, and oligomycin, an inhibitor of F1F0-ATP synthase[35]. FCCP causes a rapid depletion of the cellular bulk ATP pool, but this process is not observed if oligomycin (1 μmol/L) is applied[36]. FCCP (300 nmol/L)/oligomycin (1 μmol/L) terminated cytoplasmic Ca^2+^ transients and spontaneous activity (Fig 3D) after 2 min. This effect was reversible by washout (data not shown). FCCP/oligomycin inhibited intracellular Ca^2+^ transients and spontaneous beating (Figs 2D, 3D and 5D). The lack of ATP depletion during this timeframe was confirmed as shown in the Supplementary Material.

Reduced mitochondrial Ca^2+^ sequestration could decrease mitochondrial Ca^2+^ release, reduce the activity of sarcolemmal NCX, and lead to reduced automaticity. The mitochondrial NCX blocker, 3 μmol/L CGP, terminated AP firings in RYR2^-/-^ CMs (Fig 3D) within 2 min, which is consistent with the previous report in HL-1 CMs[12]. This result suggested that mitochondrial Ca^2+^ efflux was an indispensable step for automaticity when SR Ca^2+^ handling is depressed. At the same time, cytoplasmic Ca^2+^ handling was totally inhibited by CGP (Fig 5D). Confirming the requirement for sarcolemmal NCX in spontaneous activity, the sarcolemmal NCX1 specific blocker, KB-R7943 (5 μmol/L), could terminate CM electrical firing and cytosolic Ca^2+^ transients in RYR2^-/-^ CMs (Fig 3B and Fig 5B).

The features of APs and cytoplasmic Ca^2+^ transients recorded in Wt and RYR2^-/-^ mouse ESC-derived ventricular-like CM are listed in Table 1 and Table 2. Compared to Wt CMs, RYR2 knockout (RYR2^-/-^) CMs showed a smaller AP amplitude, a shorter AP duration, a more depolarized minimum diastolic membrane potential, a smaller cytoplasmic Ca^2+^ transient amplitude, a slower rate of rise of the Ca^2+^ transient, and a slower Ca^2+^ decay rate. The basal Ca^2+^ concentrations were similar, however.

**Table 1:** The characteristics of APs recorded in Wt and RYR2^-/-^ ventricular-like CM.

	Wt	n	RYR2^-/-^	n
**APD90 (ms)**	279 ± 25	9	196 ± 11*	6
**APA (mV)**	115 ± 4	9	92 ± 3*	6
**RMP (mV)**	-80.4 ± 2.6	9	-66.3 ± 2.0*	6

AP: action potential. APD90: AP duration at 90% repolarization. APA: AP amplitudes. RMP: resting membrane potentials. n: number of cells in each group. Data were presented as mean ± SE.

*p<0.05 compared with that in WT group.

**Table 2:** The parameters of cytoplasmic Ca^2+^ transients recorded in Wt and RYR2^-/-^ ventricular-like CM.

	Amplitude (F/F_0_)	Baseline (F/F_0_)	time to 90% peak (ms)	TD50 (ms)
**Wt Control**	0.74 ± 0.09	1.23 ± 0.03	52 ± 1	211 ± 19
**Wt Ryanodine**	0.40 ± 0.05^†^	1.14 ± 0.02	189 ± 3^††^	741 ± 73^†^
**Wt 2-APB**	0.19 ± 0.01^††^	1.09 ± 0.01	92 ± 5^†^	326 ± 13^†^
**RYR2^-/-^ Control**	0.38 ± 0.02*	1.25 ± 0.03	192 ± 3**	613 ± 43*
**RYR2^-/-^ Ryanodine**	0.33 ± 0.02	1.17 ± 0.03	196 ± 4	601 ± 19
**RYR2^-/-^ 2-APB**	0.29 ± 0.03	1.24 ± 0.02	254 ± 6*	615 ± 23*

SR Ca^2+^ ryanodine receptor blocker, 10 μmol/L Ryanodine, and the IP_3_R blocker, 2 μmol/L 2-APB, were used.TD50: transient duration at 50% amplitude. The number of cells in Wt Control, Wt Ryanodine, Wt 2-APB, RYR2^-/-^ Control, RYR2^-/-^ Ryanodine and RYR2^-/-^ 2-APB groups are 28, 21, 26, 21, 15 and 19 respectively. Data are presented as mean ± SE.

*p<0.05

**p<0.01 compared with that in WT group.

^†^*p*<0.05

^††^*p*<0.01 compared with that in control group.

Beating frequency was significantly slowed down by 2 μmol/L 2-APB from 1.3 ± 0.1 to 0.6 ± 0.1 Hz (*p*<0.05, Fig 3C and 3E). In RyR^-/-^ ESC-derived CMs, the average beating rate was 1.39 ± 0.15 Hz and decreased to 0.52 ± 0.20 Hz after 2 μmol/L 2-APB was applied, suggesting that both SR Ca^2+^ release pathways are involved in generating spontaneous electrical activity. Cytoplasmic Ca^2+^ transients were similarly affected (Fig 5C). Inhibition of IP_3_Rs by 2-APB did not decrease the beating rate further when MCU was silenced in ESC-derived ventricular-like RYR2^-/-^ CMs (Fig 6D, 6E and 6F).

Our experiments show that mitochondrial Ca^2+^ cycling can influence spontaneous electrical activity of ventricular-like CMs, especially in states of reduced SR Ca^2+^ release. Blocking mitochondrial Ca^2+^ uptake pharmacologically or by knockdown resulted in reduced spontaneous beating. The effect was more pronounced in RYR2^-/-^ CMs. Eliminating all mitochondrial Ca^2+^ flux in the absence of RYR2 prevented automaticity entirely, suggesting mitochondrial Ca^2+^ flux helps sustain spontaneous beating when SR Ca^2+^ release is inhibited. Reduced SR Ca^2+^ release and increased mitochondrial Ca^2+^ efflux are hallmarks of heart failure[29], implying that mitochondria may play a more prominent role in automatic rhythms occurring in heart failure.

Under normal conditions, SR Ca^2+^ release through RYR2 appears to play a modulatory role in spontaneous beating. Despite electrical remodeling with RYR2 knockout, eliminating SR Ca^2+^ release through RYR2 either pharmacologically or by knockout, resulted in slowing but not elimination of automaticity. The L-type Ca^2+^ channel and RYR2 form a tightly coupled pair when influencing spontaneous beating. This is consistent with other reports[12].

SR IP_3_Rs also appeared to have a significant role in modulating spontaneous beating. Blocking IP_3_R in the presence or absence of RYR2 slowed spontaneous beating. Knockdown of the mitochondrial Ca^2+^ uptake pathway, eliminated the effect of IP_3_R inhibition on the spontaneous beating rate. These observations are most easily explained if SR Ca^2+^ release from IP_3_R is tightly linked to mitochondrial Ca^2+^ handling. This is consistent with previous observations that Ca^2+^ is transferred directly between endoplasmic reticulum and mitochondria[37, 38]. It is well known that Ca^2+^ influx from the L-type Ca^2+^ channel can induce a larger Ca^2+^ release from the SR. Normal cytoplasmic Ca^2+^ is then restored in part by the sarcolemmal NCX, which generates a depolarizing current. This can explain why blocking the L-type Ca^2+^ channel in ventricular-like ESC-derived Wt CMs can eliminate spontaneous beating.

### The effect of mitochondrial Ca^2+^ flux on the automaticity of hiPSC-derived CMs

**Fig 7:**
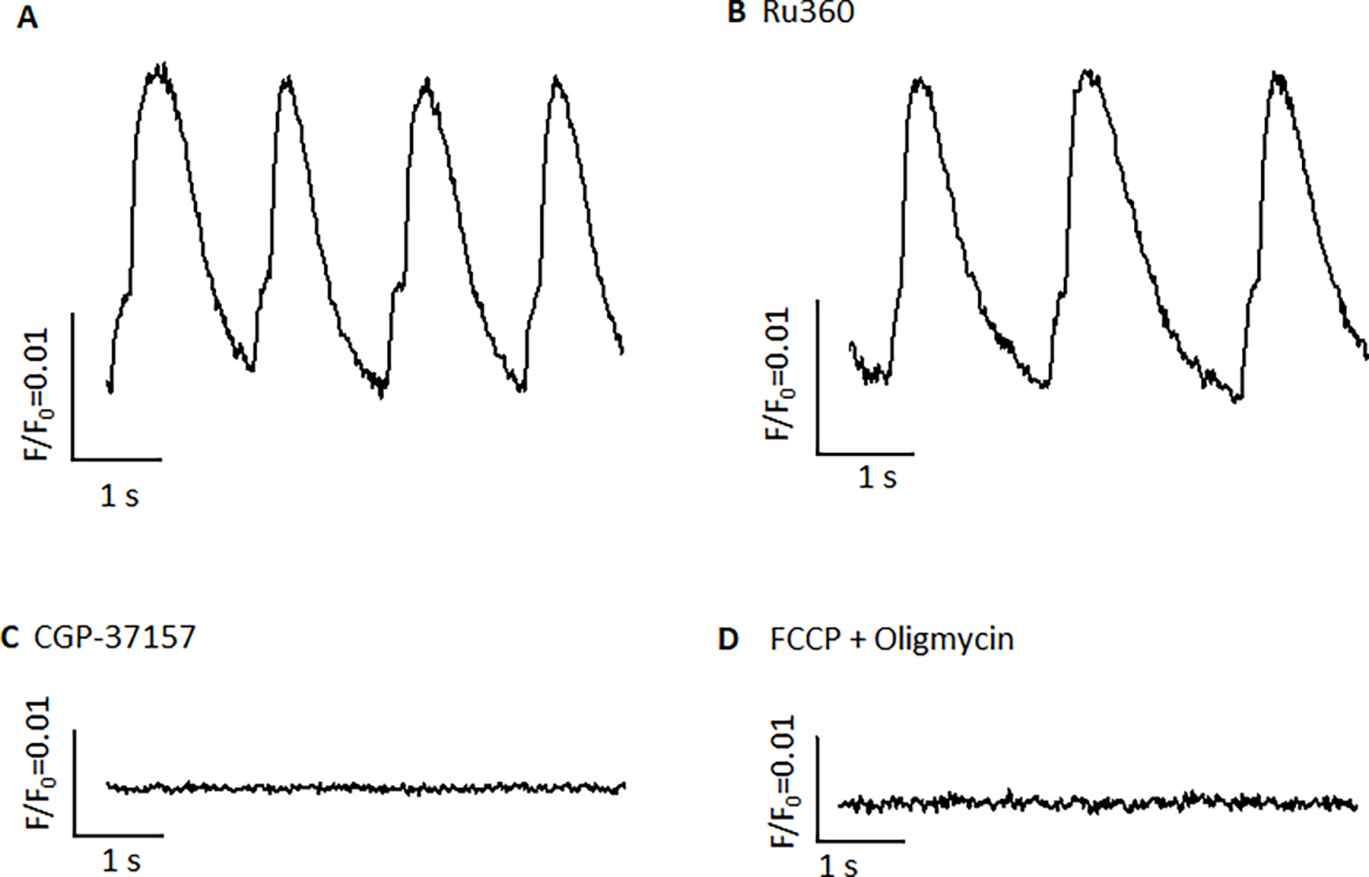
The effect of mitochondrial Ca^2+^ flux on spontaneously beating in hiPSC-derived ventricular-like CMs. **A**) Typical cytoplasmic Ca^2+^ transients in a hiPSC-derived CM. **B**) Inhibition effects of mitochondrial Ca^2+^ influx blocker, 1 μmol/L Ru360 (in pipette solution), on the beating rate of cytoplasmic Ca^2+^ transients. The spontaneous beating rate is reduced by 21.2 ± 4.3% (n = 10). **C**) The mitochondrial NCX (NCLX) blocker, 3 μmol/L CGP-37157, abolishes the automaticity of CMs (n = 11). **D**) Cytoplasmic Ca^2+^ transients are abolished by mitochondrial depolarization by 300 nmol/L FCCP and 1 μmol/L oligomycin (n = 9).

Inhibiting mitochondrial Ca^2+^ influx by 1 μmol/L Ru360 could decrease mitochondrial Ca^2+^ release, reduce the activity of sarcolemmal NCX, and reduce the rate of spontaneous beating in hiPSC-derived ventricular CMs (Fig 7A and 7B). The spontaneous beating rate was reduced by 21.2 ± 4.3% (n = 10) after Ru360 within 2–5 min of when the drug was applied. The mitochondrial NCX blocker, 3 μmol/L CGP, eliminated Ca^2+^ transients in these CMs (Fig 7C) within 2–5 min of application. Depolarization of mitochondria and preservation of ATP by the combination of 300 nmol/L FCCP and 1 μmol/L oligomycin abolished the automaticity of hiPSC-derived ventricular CMs (Fig 7D). The [ATP]_i_ level was not altered significantly during the combination of FCCP and oligomycin treatment (S1 Fig). These results were consistent with those obtained from mouse ESC-derived ventricular CMs.

### Mitochondrial Ca^2+^ flux regulated spontaneous beating in acutely isolated, adult CMs

**Fig 8:**
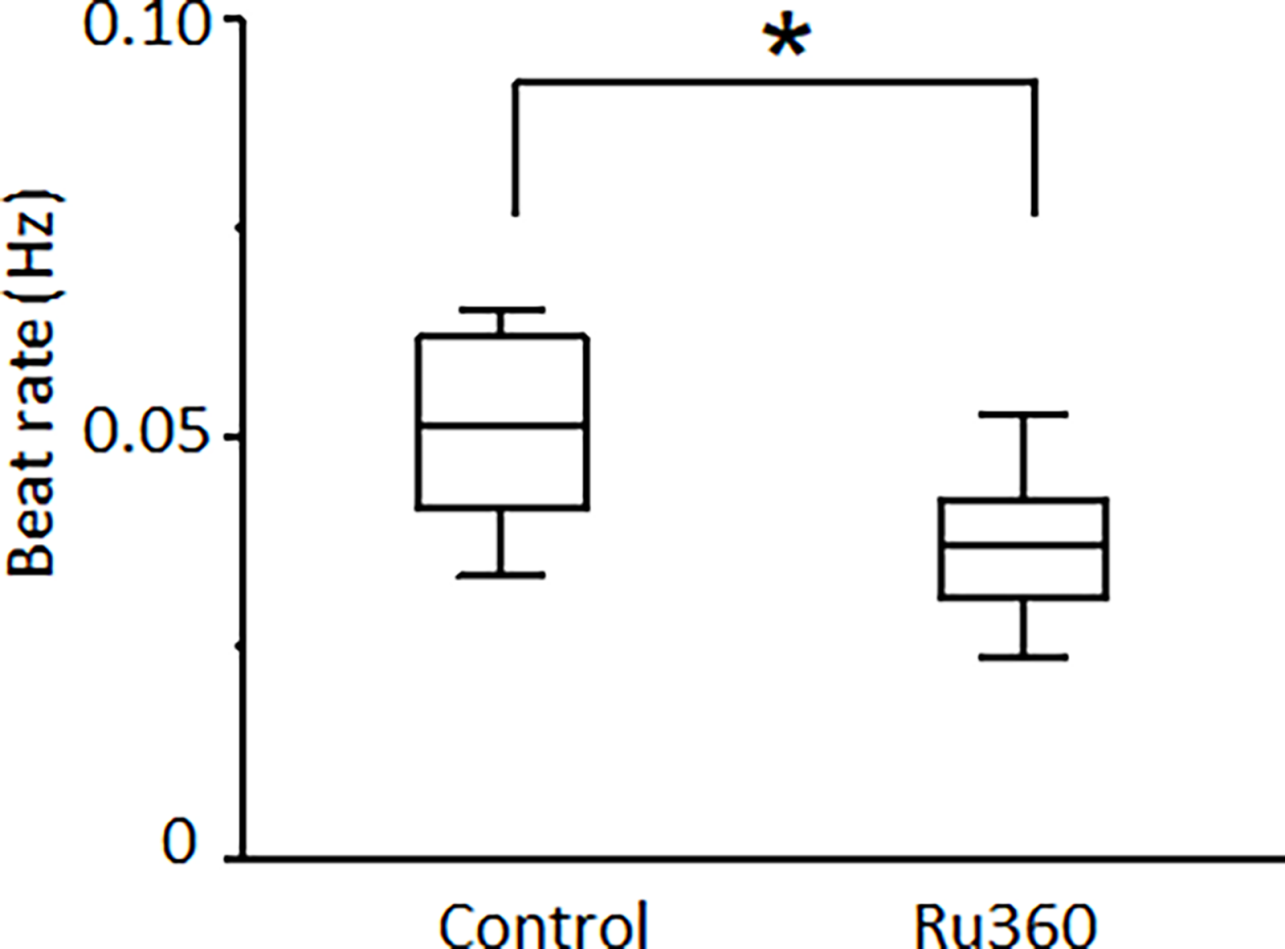
Regulation of abnormal automaticity by mitochondrial Ca^2+^ flux in ischemic mouse ventricular CMs. MCU blocker, 1 μmol/L Ru360 (in the pipette solution), reduces the ischemia-induced spontaneous beating rate of cytoplasmic Ca^2+^ transients in mouse ventricular CMs (*p<0.05, compared with control groups. n = 11).

Mouse ES cell-derived ventricular CMs were chosen because of their stable nature in culture, the presence of spontaneous beating, and genetic tractability. Human iPSC-derived ventricular CMs were used to ensure the results applied across species. Now, we have added experiments with acutely isolated, adult mouse ventricular cardiac myocytes, showing that the results apply to mature cardiac cells. Unlike the ESC-derived or hiPSC-derived ventricular CMs, normal adult mouse ventricular CMs do not show spontaneous electrical activity[39].Nevertheless, abnormal automaticity can be elicited in matured ventricular CMs by acute ischemia[40]. In this study, simulated ischemic buffer was used to induce spontaneous beating in isolated adult mouse ventricular cells. Inhibiting mitochondrial Ca^2+^ influx by 1 μmol/L Ru360 could significantly reduce the beating rate from 0.051 ± 0.003 Hz to 0.037 ± 0.003 Hz in mouse ventricular CMs (Fig 8).

**Fig 9:**
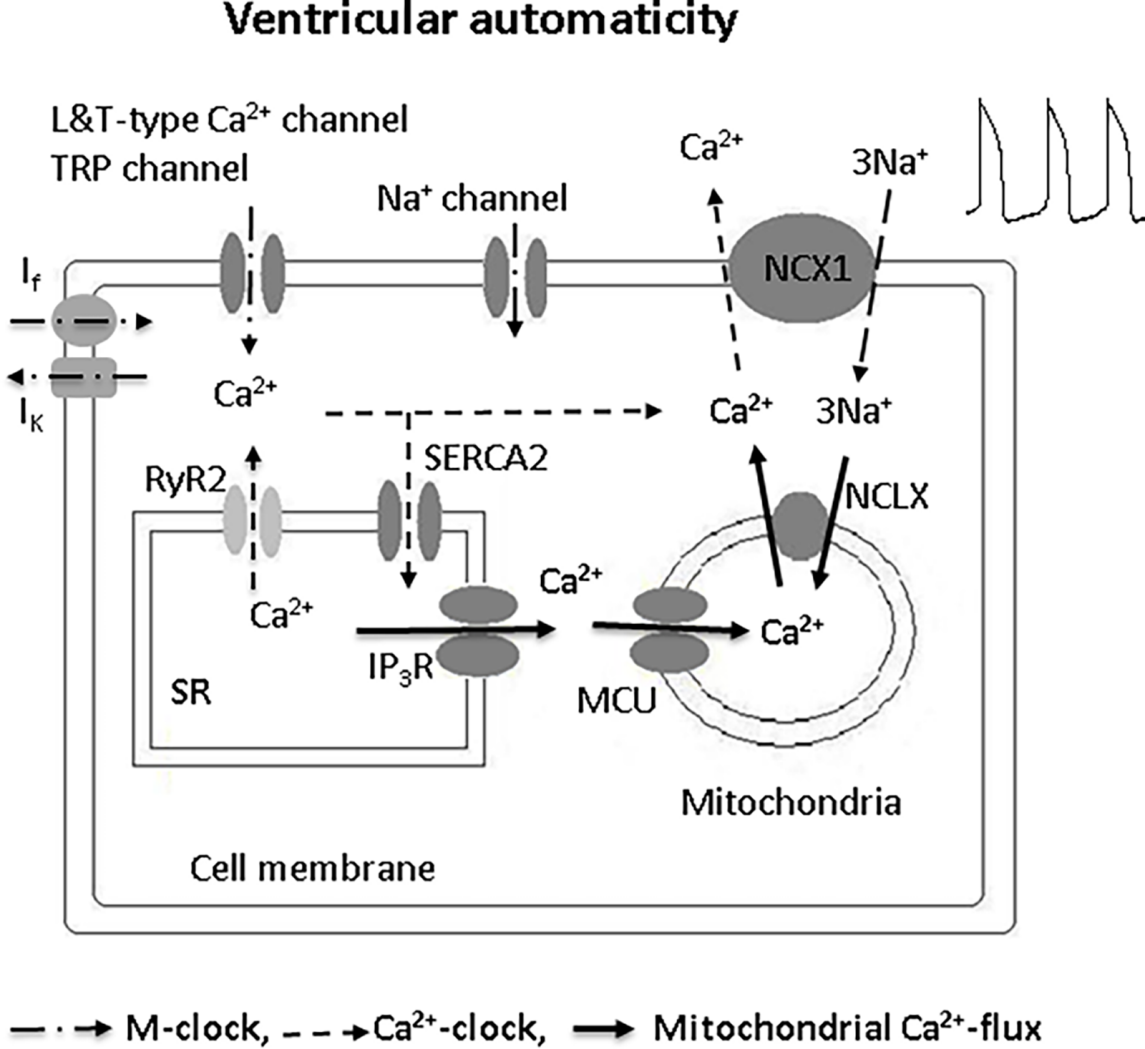
Proposed model of ion channels and transporters modulating spontaneous beating in ventricular-CMs. Sarcolemmal and mitochondrial NCXs are necessary for spontaneous beating. The mitochondrial membrane potential, L-type Ca^2+^ channels, and Na^+^ channels are also required. NCLX: mitochondrial NCX. NCX1: NCX type 1. Solid lines represent mitochondrial Ca^2+^ flux pathways for automaticity. Dashed lines show Ca^2+^ clock and dash dot line is M-clock (Voltage clock).

The electrophysiology of ventricular-like ESC-derived or hiPSC-derived CM may not recapitulate exactly the electrophysiology of intact ventricular cells. For example, they have reduced I_K1_ and increased I_f_ when compared to acutely isolated ventricular cells[20]. On the other hand, our findings that MCU inhibition alters spontaneous beating in adult ventricular CMs reinforces our central finding that mitochondrial Ca^2+^ handling plays a role in spontaneous electrical activity. This result is consistent with previous studies showing a role for mitochondrial Ca^2+^ handling in pacemaker/atrial cell automaticity and extends these results to ventricular cells. In the case of pacemaker cells, inhibition of MCU or the mitochondrial NCX had opposing effects on beating rate, with MCU inhibition accelerating beating. These effects were ascribed to a mitochondrial Ca^2+^ buffering effect that ultimately led to mitochondrial ATP production changes that balanced the metabolic needs of SR Ca^2+^ flux. In the case of ventricular-like CMs, automaticity was influenced differently from pacemaker cells by changes in mitochondrial Ca^2+^ handling, and inhibition of MCU or mitochondrial NCX had similar effects to reduce beating rate. Based on our data, it is reasonable to speculate that reduced mitochondrial Ca^2+^ handling caused reduced NCX activity as shown in Fig 9, with the effect of mitochondrial Ca^2+^ handling changes on spontaneous beating being independent of SR Ca^2+^ handling. Whether the influence of mitochondrial Ca^2+^ flux was independent of the M-clock or metabolic demand needs further study. Our findings suggest that the role of the mitochondria in spontaneous beating differs between pacemaker cells and ventricular-like CMs. The fact that mouse and human CMs showed similar responses suggests that the role of mitochondria in spontaneous beating is species independent and relevant to the human condition.

The state of mitochondrial maturity in our experiments is unknown. Nevertheless, the experiments show proof of principle that mitochondrial Ca^2+^ flux may contribute to spontaneous beating and possibly arrhythmic risk. In support of this idea, SR Ca^2+^ release in adult mouse ventricular myocytes contributes 90% of the Ca^2+^ transient [41]. The contribution of the SR Ca^2+^ flux to the Ca^2+^ transient was 72% in our ventricular-like ESC-derived CMs [8]. The later contribution is close to that in adult mouse ventricular cells after myocardial infarction[42]. Therefore, the effect of mitochondrial flux on spontaneous beating in adult mouse ventricular cells was studied in simulated ischemia condition.

MCU activity is increased in ischemia and cardiomyopathy[43, 44]. Although none of these studies looked at arrhythmic risk, based on our results, the increased MCU activity in these disease states might be expected to enhance spontaneous beating in ventricular cells. Therefore, inhibiting MCU activity might reduce the risk of arrhythmias based on the expected enhanced or abnormal automaticity.

## Conclusions

Spontaneous beating in ventricular cells involves mitochondrial Ca^2+^ flux and sarcolemmal NCX currents. RYR2/L-type Ca^2+^ channel and IP_3_R/MCU pairs appear to form functional units of Ca^2+^ handling. These results suggest that mitochondria may influence ventricular spontaneous depolarization and abnormal automaticity by altering mitochondrial Ca^2+^ handling (Fig 9).

## Supporting information

S1 Fig Intracellular free ATP level was not altered significantly during the combination of FCCP and oligomycin treatment. The average effect of FCCP (w/wo oligomycin) on [Mg^2+^]_i_ in hiPSC-derived ventricular-like CMs (n = 10 for each group).
(PDF)
