# Supplementary material for: Mitochondrial Ca2+ flux modulates spontaneous electrical activity in ventricular cardiomyocytes
Source: PLoS One. 2018 Jul 12;13(7):e0200448. doi: 10.1371/journal.pone.0200448 (PMC6042741; doi:10.1371/journal.pone.0200448)

MATERIALS AND METHODS

Intracellular Mg^{2+} measurement

Free intracellular Mg^{2+} concentration ($[Mg^{2+}]_i$) was labeled by Mag-Fluo-4 AM (ThermoFisher Scientific). The hiPSC-derived CMs in Tyrode solution were loaded with 10 μM Mag-Fluo-4 AM for 40 minutes at 37^oC followed by 2 washes and 30min de-esterification[1]. Mag-Fluo-4 was excited at 488nm and emitted fluorescence was collected at wavelengths of 510-560 nm. Two dimensional images (2048 × 2048 pixels) were acquired by a real-time florescence microscope (Olympus IX81, Japan) at 30 s intervals. Analysis of the signals was performed with the software MetaMorph (version 7.8.11.0, Nashville, TN). The change of $[Mg^{2+}]_i$ were presented as background-subtracted normalized fluorescence (F/F_0).

RESULTS

The effect of FCCP on $[Mg^{2+}]_i$ in hiPSC-derived ventricular-like CMs

Almost all the intracellular Mg^{2+} is bound to ATP, and only a small fraction is free. Since free $[Mg^{2+}]_i$ is kept constant within a very narrow range, any change in cellular ATP levels leads to a concomitant change in free $[Mg^{2+}]_i$. Thus, changes in $[Mg^{2+}]_i$ can be interpreted as reciprocal changes of $[ATP]_i$[2]. FCCP caused a rapid depletion of the cellular bulk ATP pool which can be reflected by an increased $[Mg^{2+}]_i$. Nevertheless, this phenomenon was not observed if oligomycin (1 μmol/L) was applied (Fig S1). This indirectly indicated that the $[ATP]_i$ level was not altered significantly during the combination of FCCP and oligomycin treatment.

REFERENCES

1. Kawahara K, Sato R, Iwabuchi S, Matsuyama D. Rhythmic fluctuations in the concentration of intracellular Mg^{2+} in association with spontaneous rhythmic contraction in cultured cardiac myocytes. Chronobiol Int. 2008;25: 868-81.
2. Zima AV, Pabbidi MR, Lipsius SL, Blatter LA. Effects of mitochondrial uncoupling on Ca^{2+} signaling during excitation-contraction coupling in atrial myocytes. Am J Physiol Heart Circ Physiol. 2013;304: H983-93.

FIGURE LEGEND

Fig S1. The average effect of FCCP (± oligomycin) on $[Mg^{2+}]_i$ in hiPSC-derived ventricular-like CMs (n=10 for each group).

Fig. S1 Intracellular free ATP level is not altered significantly during the combination of FCCP and oligomycin treatment.

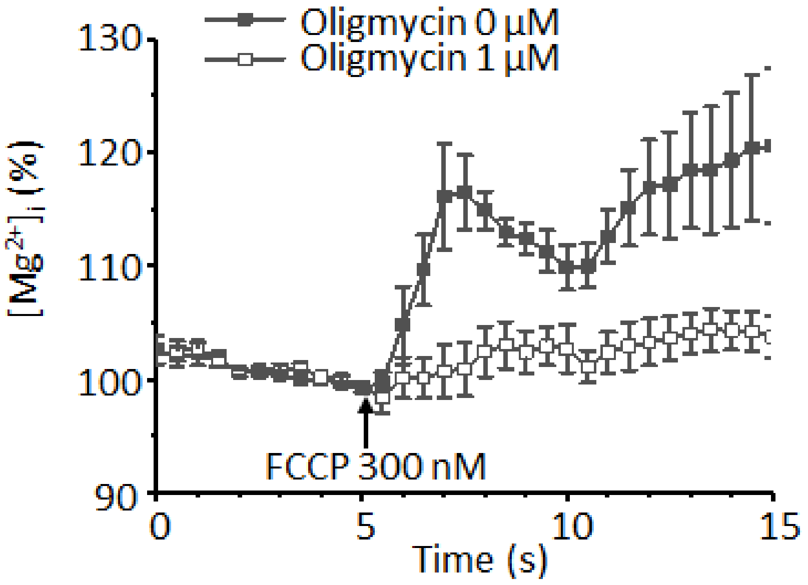

Supplement: S1 Fig — The average effect of FCCP (w/wo oligomycin) on [Mg2+]i in hiPSC-derived ventricular-like CMs (n = 10 for each group). (PDF) [file pone.0200448.s001.pdf]
